# Conditions and strategies influencing sustainability of a community-based exercise program incorporating a healthcare-community partnership for people with balance and mobility limitations in Canada: A collective case study of the Together in Movement and Exercise (TIME™) program

**DOI:** 10.3389/fresc.2023.1064266

**Published:** 2023-02-27

**Authors:** Gayatri Aravind, Ian D. Graham, Jill I. Cameron, Michelle Ploughman, Nancy M. Salbach

**Affiliations:** ^1^ Department of Physical Therapy, University of Toronto, Toronto, ON, Canada; ^2^ March of Dimes Canada, Toronto, ON, Canada; ^3^ Ottawa Hospital Research Institute, Ottawa, ON, Canada; ^4^ Department of Occupational Science and Occupational Therapy, Centre for Practice Changing Research, University of Toronto, Toronto, ON, Canada; ^5^ The KITE Research Institute, University Health Network, Toronto, ON, Canada; ^6^ BioMedical Sciences, Faculty of Medicine, Memorial University of Newfoundland, St. John’s, NL, Canada

**Keywords:** community exercise, balance, mobility, implementation, sustainability, case study, healthcare-community partnerships, qualitative research

## Abstract

**Background:**

Community-based exercise programs delivered through healthcare-community partnerships (CBEP-HCPs) are beneficial to individuals with balance and mobility limitations. For the community to benefit, however, these programs must be sustained over time.

**Purpose:**

To identify conditions influencing the sustainability of CBEP-HCPs for people with balance and mobility limitations and strategies used to promote sustainability based on experiences of program providers, exercise participants, and caregivers.

**Methods:**

Using a qualitative collective case study design, we invited stakeholders (program providers, exercise participants, and caregivers) from sites that had been running a CBEP-HCP for people with balance and mobility limitations for ≥4 years; and sites where the CBEP-HCP had been discontinued, to participate. We used two sustainability models to inform development of interview guides and data analysis. Qualitative data from each site were integrated using a narrative approach to foster deeper understanding of within-organization experiences.

**Results:**

Twenty-nine individuals from 4 sustained and 4 discontinued sites in Ontario (*n* = 6) and British Columbia (*n* = 2), Canada, participated. Sites with sustained programs were characterized by conditions such as need for the program in the community, presence of secure funding or cost recovery mechanisms, presence of community partners, availability of experienced and motivated instructors, and the capacity to allocate resources towards program marketing and participant recruitment. For sites where programs discontinued, diminished participation and/or enrollment and an inability to allocate sufficient financial, human, and logistical resources towards the program affected program continuity. Participants from discontinued sites also identified issues such as staff with low motivation and limited experience, and presence of competing programs within the organization or the community. Staff associated the absence of referral pathways, insufficient community awareness of the program, and the inability to recover program cost due to poor participation, with program discontinuation.

**Conclusion:**

Sustainability of CBEP-HCPs for people with balance and mobility limitations is influenced by conditions that exist during program implementation and delivery, including the need for the program in the community, and organization and community capacity to bear the program's financial and resource requirements. Complex interactions among these factors, in addition to strategies employed by program staff to promote sustainability, influence program sustainability.

## Introduction

Over the past decade, community-based exercise programs (CBEPs) have emerged to meet the demand for accessible and supervised exercise programs for individuals with balance and mobility limitations within their own communities (1). For people with stroke, participating in CBEPs has the potential to improve balance, mobility and functional independence, and reduce the risk of a recurrent stroke and secondary complications related to a sedentary lifestyle (2–4).

Not all CBEPs are informed by research evidence. CBEPs that integrate a novel healthcare-community partnership (CBEP-HCP) take advantage of complementary missions, infrastructure, and expertise of healthcare and recreation organizations to deliver an evidence-informed community-based exercise program (5). In this model, a healthcare partner (e.g., physical therapist, kinesiologist) is engaged to train and support fitness instructors to deliver a task-oriented exercise program targeting balance and mobility in community settings (5). Globally, various models of CBEP-HCP deliver programs to people with stroke such as the Adapted Physical Activity Program (6–8), a cycling program (9), Fit For Function (10), Fitness and Mobility Exercise (FAME) (11–13), Graded Repetitive Arm Supplementary Program (GRASP) (14), Neurological Exercise Training (NExT) (15), Together in Movement and Exercise (TIME™) (1, 5, 16), and Rehabilitation Training (ReTrain) (17).

Effective programs must be sustained to ensure continued access and benefit for community-dwelling people with balance and mobility limitations (18–21). Not all programs, however, are successful in the longer term and, in most cases, the reasons for discontinuation are not reported (22). Understanding factors that contribute to the success or failure of CBEP-HCPs is critical to ensuring future success.

The elements of program sustainability can be narrowed down to three domains: factors that exist within the organization implementing the program; factors that exist outside of the organization (broader community); and program features. Shediac–Rizkallah and Bone (23) proposed that exploring these three groups of factors allows for broad and open interpretation of sustainability. Using this model, Mancini and Marek (24) created a program sustainability index which identifies the individual factors within these three silos. Thus, combining the Shediac–Rizkallah and Bone (23) and the Mancini and Marek (24) models provides a structure enabling the exploration of factors governing sustainability at broader and granular levels.

In previous research, factors contributing to the sustainability of government- or research–funded health initiatives in the areas of mental health (25), cancer screening (26), substance abuse (27), smoking cessation (28), nutrition (29), and health promotion (29, 30), have been examined. Little attention has been paid to the sustainability of CBEP-HCPs in real world settings. Therefore, the primary objective of this study was to identify conditions influencing the sustainability of CBEP-HCPs and strategies implemented to promote sustainability based on experiences of users involved in program implementation and delivery, and exercise participants and their caregivers. A secondary objective was to develop a checklist of questions to aid organizations with sustaining CBEP-HCPs.

## Materials and methods

### Study design

We used a qualitative collective case study design to understand the experiences of key stakeholders involved with sites that sustained or discontinued a licensed CBEP-HCP called the Together in Movement and Exercise (TIME™) program (31). A case study approach is well-suited to study program sustainability as it permits in-depth exploration of a complex issue within a real-life context (32). We chose a collective case study design as it involves examination of multiple cases at a time to generate a broad understanding of the variation in experiences across “cases” (33). We used the Consolidated criteria for reporting qualitative research (COREQ) (34) to guide reporting. The University of Toronto health sciences research ethics board approved the study protocol.

### Case selection

We selected the TIME™ program for the exploration of sustainability as developers have maintained records of program implementation, delivery and discontinuation. Following a demonstration of safety, feasibility, and potential benefit (5), the TIME™ program was adopted by more than 50 sites across Canada. Between 2008 and 2017, however, at least 15 centres discontinued the TIME™ program (personal communication with TIME™ program developers) for reasons that were not well understood.

### Together in movement and exercise (TIME™) exercise program

The TIME™ program is a task-oriented, group exercise program designed to improve independence in everyday functioning among individuals with balance and mobility limitations (5). Trained fitness instructors, who are supported by healthcare partners [typically physical therapists (35)] and volunteers, deliver the program. The program relies on a partnership between a community organization that hosts the program and provides the resources (e.g., instructors, space, equipment, volunteers) and a healthcare partner (from a local hospital or community practice) who visits classes and serves as a resource for the instructors and supports participant enrollment directly by referring participants, or by creating referral pathways from local sources of participants (5, 22, 35). Other individuals involved in program implementation and delivery include recreation managers who are involved in decisions about whether or not to implement a program in the organization and program coordinators who oversee the program planning and allocation of resources. In some organizations the same individual may fulfil these roles (36).

### Sampling and eligibility

The sampling frame consisted of a list of 52 sites with a license to offer the TIME™ program, including 37 sites currently offering the TIME™ program, and 15 sites that had discontinued the TIME™ program (as of April 2019). In consultation with TIME™ developers and members of the Canadian Advisory Collaborative for TIME™ (CAN-ACT), we developed a list of 8 sustained programs that had been running for at least 4 years and whose team would be potentially willing to share their experiences. Examining multiple cases spanning the spectrum of organizational identities, priorities and experiences was expected to provide a better understanding of the drivers and barriers of CBEP-HCP program sustainability. Similarly, we created a list of 8 discontinued programs where individuals who were involved with the program were still available and would potentially be willing to share their experiences. Each case was selected for its similarities or differences with respect to the outcome of interest (i.e., program sustainability) – ensuring theoretical replication, where the outcomes are contradictory (failed vs. successful sites) (33). Such comparative case studies are used to identify how features within the context influence the success of programs (33, 37).

Sites were considered eligible if they held a valid license from the University Health Network (Toronto, Canada) to run the TIME™ program, and fitness instructors that had been trained to deliver the TIME™ program. A sustained site had to meet the following additional criteria: currently offering or delivering the TIME™ program; have a healthcare partner; have delivered the TIME™ program at least once per year for the past four years; and intend to offer the TIME™ program for the next two years. A discontinued site had to meet the following criteria: have delivered at least one TIME™ program; discontinued offering the TIME™ program; have no plans to resume offering the TIME™ program.

### Participant eligibility and recruitment

Information regarding the organization, its priorities, resources, and the processes involved can be best obtained from stakeholders involved in the organization, program delivery and program utilization (38). In the case of the TIME™ program, stakeholders included program managers, coordinators, instructors, volunteers, healthcare partners, program participants and their caregivers. Within each site, we aimed to recruit at least one participant for each stakeholder group based on the eligibility criteria detailed in Table 1. Use of a qualitative approach involving interviews with these stakeholders allowed us to obtain a detailed account of their experiences with the program for each case (site) (39, 40).

**Table 1:** Eligibility criteria.

Participant Group	Eligibility criteria
TIME™ program participant	A participant of the Together in Movement and Exercise (TIME™) program with balance and mobility limitations was eligible to participate. Any individual with apparent speech or cognitive difficulties subsequent to the stroke, who was unable to comprehend questions or convey their perspectives, was excluded.
Caregiver of TIME™ participant	A paid or unpaid caregiver who assisted the TIME™ program participant to live independently at home and provided support and assistance with basic and/or instrumental activities of daily living at least once a week was eligible
Fitness instructor	A trained individual that had delivered at least one TIME™ program.
Volunteer	A trained individual that assisted with TIME™ program delivery.
Recreation coordinator	An individual involved in the day-to-day management and supervision of fitness instructors delivering the TIME™ program.
Program manager	An individual at each community centre involved in the decision to implement the TIME™ program, and TIME™ program management and oversight.
Healthcare partner	A healthcare professional involved with training the fitness instructors to deliver the TIME™ program and providing ongoing guidance and support through the TIME™ program, with experience of at least one TIME™ program.

Sites were recruited *via* emails to the recreation manager or program coordinator. In sites where managers/coordinators agreed to participate, they were asked to identify and connect the lead researcher (GA) with at least one individual who was involved with the program as an instructor, healthcare partner, participant, caregiver, and volunteer, and expressed interest in knowing more about the study. The lead researcher contacted interested participants *via* telephone and obtained verbal informed consent from individuals who agreed to participate.

### Data collection

Sustainability models by Shediac–Rizkallah and Bone (23) and Mancini and Marek (24) were used to develop interview guides tailored to each group. In keeping with the models, the interview guide included questions to explore the outer and inner contexts, and the influences of the program design on program implementation. For the purpose of this study, the inner context for the program was determined by the organizational and managerial structures and processes that support or pose challenges to program continuity. The outer setting included the community at large that was served by the program and is influenced by the political, economic and social environment within which the program functioned. Finally, the program design constituted human, logistical, and financial resources utilized and made available to the program for set-up, delivery, and continuation as well as the plan of action executed to implement the program. Table 2 presents sample interview questions. The interview guide was pilot tested with a fitness instructor with previous experience delivering the TIME™ program, and a previous participant of the TIME™ program. A single interviewer (GA), a cis-gender female physical therapy researcher with a PhD in rehabilitation science, 8 years of experience in stroke-related research, and 2 years of experience with qualitative research, completed one-on-one semi-structured interviews by telephone. Prior to beginning the recording, the interviewer shared their background, experience, and their academic interest in understanding program sustainability. The interviewer was unknown to the participants prior to the study. The participants attended the interview from their homes while the interviewer conducted the interview from a private workspace. Interviews lasted 45–60 min. Interviews were audio-recorded and professionally transcribed. The lead researcher (GA) and a trained research assistant (RA) verified transcripts for accuracy by comparing them to the audio-recordings.

**Table 2:** Sample questions from interview guide for program coordinator.

Prior to the TIME™ Program, did the centre provide exercise or recreation programs for people with balance and mobility limitations due to stroke or other chronic diseases?
The TIME™ program is for people with balance and mobility problems related to stroke and other chronic conditions. What were the reasons to implement the program in your organization?
What challenges did you encounter in the process of (implementing the TIME™ program)? How did you resolve or overcome these challenges?
Where do participants typically come from? Who do you partner with? What additional outreach or marketing do you do? What are your primary referral sources?
What are the other ways in which you attract participants to the TIME™ program?
Are there other recreation centres serving the same geographical region, offering programs similar to the TIME™ program for people with balance and mobility limitations?
How does the presence of competing programs affect the TIME™ program? How does this influence your decision to continue to offer the TIME™ program at your centre?
Once the program is implemented, who is involved in the decision making process when deciding to continue or discontinue offering a program? What do you consider during such evaluations? How often do you evaluate your decision to continue to offer a program?
In your opinion, what are the key factors that determine the success [or failure] of long-term program sustainability?
Who are the key stakeholders that determine the success [or failure] of long-term program sustainability?

### Data analysis

De-identified transcripts were analyzed using a directed content analysis approach (41) and included deductive and inductive coding. The lead researcher (GA) developed a codebook *a priori*, which was reviewed by two members of the research team (NMS, JIC), based on the primary objective and the factors described in the two sustainability models. Two researchers (GA, NMS) independently reviewed and coded transcripts from one site and met to discuss codes. Based on this review, new codes were developed to capture information that could not be coded using existing codes based on the sustainability frameworks (codebook available on request). Then the lead researcher (GA) and the RA coded the remaining transcripts using NVivo 12. Data for each site were analyzed separately to develop case summaries. First, the lead researcher reviewed node reports and created within-site summaries that outlined the context of the setting, the mechanisms by which the program was implemented, and the description of how the sites continued to deliver the program or conditions that ultimately led to program discontinuation. Second, members of the research team (GA, NMS, IDG) met to review case summaries and discuss emerging themes and key concepts. Third, the lead researcher compared and contrasted the case summaries within the sustained and discontinued categories, and then across all cases. Conditions identified as influencing sustainability and actions taken to resolve challenges and ensure sustainability were compared across sustained and discontinued cases. An inductive approach was used to identify overlap in conditions and strategies across the sites. Finally, based on the recommendations and reflections of the study participants, the team created a list of questions that existing and new programs can use to guide program implementation and sustainability planning.

### Strategies to ensure methodological rigor

Trustworthiness was enhanced by the development of a detailed research protocol and maintenance of an audit trail that allowed for transparency of the methods used to arrive at the conclusion (42, 43). The use of multiple sources of data within each case to obtain a holistic perspective improves the credibility of findings. A detailed description of each individual case (i.e., “a thick description”) provides information regarding the context within which the results have been obtained allowing for transferability of findings to other organizations (43).

## Results

### Site and participant characteristics

Twenty-eight individuals from programs at eight unique sites (four sustained and four discontinued; located in Ontario (*n* = 6) and British Columbia (*n* = 2)) participated in 27 interviews between April and August of 2019. Participants included 6 program coordinators, 8 fitness instructors, 3 program managers, 1 healthcare partner, 1 regional stroke coordinator/healthcare partner, 6 TIME™ participants, and 3 caregivers of TIME™ participants. Duration of participant involvement with the program ranged from 6 months to 8 years. Table 3 presents the characteristics of sites and participants.

**Table 3:** Site characteristics (*n* = 8).

Characteristic	Sites with sustained programs				Sites with discontinued programs			
	Site A	Site B	Site C	Site D	Site E	Site F	Site G	Site H
Population (44)	130,000	160,000	140,000	140,000	21,000	92,000	22,000	330,000
Type of center (44)	Large urban center	Large urban center	Large urban center	Large urban center	Small suburban center	Medium urban center	Small suburban center	Large urban center
Community demographics	Majority working-age adult population (<65 years old), with largest subgroup aged 50–55 years	Majority working-age adult population (<65 years old), with largest subgroup aged 50–55 years	Majority working-age adult population (<65 years old), with largest subgroup aged 30–35 years	Majority working-age adult population (<65 years old), with largest subgroup aged 50–55 years	Majority working-age adult population (<65 years old), with largest subgroup aged 65 + years	Majority working-age adult population (<65 years old), with largest subgroup aged 50–55 years	Majority working-age adult population (<65 years old), with largest subgroup aged 65 + years	Majority working-age adult population (<65 years old), with largest subgroup aged 50–55 years
Type of organization	Non-profit	Non-profit	Municipal	Municipal	Non-profit	Municipal	Non-profit	Municipal
Funding for organization	Mixed^a^	Public	Public	Public	Mixed^a^	Public	Mixed^a^	Public
Age of organization^b^	7 + years	40 + years	14 + years	52 + years	8 + years	35 + years	8 + years	7 + years
Mission of organization	To enhance the quality of life and citizenship for people of all ages and abilities by providing inclusive programs and services of the highest quality and value	To provide assistance to live independently and inclusively in the community through individualized support and rehabilitation services	To continually improve the quality of life within the community through key services for current and future generations	To provide access to barrier-free recreation for all ages and abilities within the municipality	The organization is dedicated to the growth of all persons in spirit, mind, and body, and to their sense of belonging to each other and the global community	The organization is guided by the following values: respect, honesty, enthusiasm, progressiveness and community responsibility	The organization is dedicated to the growth of all persons in spirit, mind, and body, and to their sense of belonging to each other and the global community	To provide inclusive and accessible recreation for citizens of all backgrounds and abilities
Program duration	6 years	8 years	8 years	6 years	5 years	2 years	4 years	1.5 years
Program cost (CAD)	$45 per 1-month membership/∼$280/program	Free	$11–13 per class	$100–120 per 5–6-week program	$50 per 12-week program	$80 per 12-week program	$21–25 per 2-week membership	$105 per 8-week program
Financial aid available	Yes	Not applicable	Yes	Yes	Yes	Yes	Yes	Yes
# Participant spots per class	12	8	9	10	12	10	12	8
# Fitness instructors per class	3	2	2	2	1	2	1	1
Use of volunteers (Yes/No)	Yes	No	No	Yes	Yes	No	Yes	No
# Programs/y	3	4	4	3	2–3	3	2	4
Stakeholders interviewed (time with program^b^)	1 PC (3 years)	1 PC (3 years)	1 PC (8 years)	1 PC (1 year)	1 PC (5 years)	1 PM (2 years)	1 FI (4 years)	1 PC (3 years)
	1 FI (4 years)	1 FI (8 years)	1 FI (4 years)	1 FI (2 years)	1 FI (4 years)	1 FI (2 years)	1 P (4 years)	1 FI (2 years)
	1 RSC (6 years)^c^	1 PM (5 years)	1 HCP (8 years)		1 PM (2 years)	1 RSC and HCP^c^	1 RSC and HCP^c^	
	2 P & CG dyads (6 months-1 years)	1 P (7 months)	1 P (4 years)		1 P (6 months)			
			1 CG (4 years)					

PC, program coordinator; FI, fitness instructor; RSC, regional stroke coordinator; P, participant; CG, caregiver; PM, program manager; y, years; mos, months; #, number of.

^a^ Funded through government grants, program registration, memberships, and charitable donations.

^b^ Estimated at the time of interview.

^c^ Position performed by same individual.

Based on the experiences of the participants from the sustained and discontinued programs, we identified 10 conditions that influenced the sustainability of the program. We defined conditions as the organizational, community and human factors that existed at the time of program implementation and delivery and influenced it either individually or through interaction. Table 4 describes each condition (and subcondition) with supporting quotes and the influence of each condition on sustainability at the sustained and discontinued sites.

**Table 4:** Conditions influencing TIME™ program sustainability across sites and supporting quotes.

Condition Subcondition	Description of Condition	Presence of Condition across Sites							
		A	B	C	D	E	F	G	H
1. **Alignment of program and organizational objectives**	The intended goal of the TIME™ program aligned with the organizational values, mission, or priorities. Having been designed for people with balance and mobility limitations, the TIME™ program enabled some organizations to expand their programming to new areas, while in other cases it reinforced the organization's reputation as inclusive	+	+	+	+	+	+	+	+
*“We as the municipality program (need to provide) recreation for all ages, all abilities to try to create barrier-free recreation. Implementing the TIME™ program allows individuals with limitations to come and have a safe place, a structured place, a supportive place to come and instill more movement into their lives and to take that into their outside lives and be able to be more functional.” Program Coordinator/Recreation Manager, Site D*									
2. **Economic viability**	The organization and the community were able to allocate financial, human and logistical resources towards the program with the support of dedicated funding and/or effective cost recovery mechanisms.	+	+	+	+	–	–	–	–
*Adequate enrolment*	The program experienced a continuous and regular inflow of participants directly from the community and/or through referrals or recruitment from local hospitals and healthcare centres. The number of participants enrolling into the program matched or exceeded the expectations set by program. Where applicable, program/membership fees from these participants offset the organizational costs related to program delivery.	+	+	+	+	–	–	–	–
*Stable program funding*	The program delivery was supported by a secure availability of financial resources obtained through organizational funds, financial aid from local governmental organizations, grants.	+	+	n/a	n/a	–	–	–	–
*“(Enrollment numbers are) important. I mean the [organization] is a public organization so our profit margins are quite low. I mean we do want to pull in some revenue but at the very least we need to break even with our programs. The implication would be that we’re putting in our time to teach the program but the revenues would need to make up for our time in teaching.” Fitness Instructor, Site C*									
*“We do have [amount] of funding that is permanent for us. We did work with the stroke network on that for a number of years. So we can maintain as we are now but if we would like to add more rehab support or more physiotherapy support, we do not have the funds for that.“ Program Manager, Site B*									
3. **Availability of resources**	The organization had the financial, logistical, and human resources necessary to implement and deliver the program as designed. There were no other programs within the organization competing for the same resources. The organization also had resources to create program awareness in the community, develop and nurture partnerships, and increase program intake and registration which in turn facilitated adequate enrollment.	+	+	+	+	–	–	–	–
*“Probably because the coordinators and the managers value that program and they work really hard to make sure they have the right instructors to lead it. We’re given support when we need it and the managers ran the training because they saw that there was a demand for people wanting to learn about the TIME™ program and being able to sub in it as well. So if I was sick, there are other fitness instructors that don't teach it on a regular basis but are qualified to teach it. So we have back up if we need it.” Program Coordinator, Site D*									
4. **Initial and ongoing need for program in the community**	The program filled a service gap in the community, supporting transition from hospital to home, particularly for people with stroke. It offered participants individualized attention from competent instructors that was lacking when exercising at home or the gym. It also provided opportunities for participants and caregivers to observe and interact with people with similar lived experiences which provides hope, and encouragement to continue participation. The ongoing need for a program was demonstrated by high attendance rates, current participants re-registration for future programs, and/or by the growing waitlists of participants seeking to enroll in the program.	+	+	+	+	–	–	–	–
*“With our aging population more people will be coming through this program and older people will be coming to the gym to use the equipment. They’re realizing that they need to keep their bodies moving. So it's such a great balance.” Fitness Instructor, Site D*									
*“Well obviously there's a need for this type of program as with an aging population. We do have a very large older adult population in our community and most of our successful programs that run here are older adult programs. We recognized that there is a need for it and that we’re relatively close to the [name of hospital] in [city]. We saw that as a good opportunity for networking that they could then take their patients who’d completed their rehab program and send them our way.” Fitness Instructor, Site F*									
5. **Absence of competing programs**	The program offered a unique service to the community. There were no other programs in the neighboring communities which were specifically designed for or could be accessed by individuals with balance and mobility limitations.	n/a	–^a^	n/a	–^a^	–	–	n/a	–
*“I think part of it is the size of the municipalities. When you think about [name of 2 cities], that's not a huge…I’m going to say maybe between the two areas is 30,000 people, maybe 40. But yet they ran two programs there. So really from a strategic perspective on their end that population probably would’ve been fine with one.” Healthcare Partner, Site E*									
6. **Initial and ongoing presence of supportive partnerships**	The program relied on and was supported by established networks and partnerships with individuals and organizations in the local community that assist with the continued delivery of the program. Support was observed through facilitation of a) program uptake by means of referrals, distribution of program information, or permitting the active recruitment of participants, or b) program delivery by serving as a healthcare partner to train program staff and supervise program delivery.	+	+	+	+	n/a	n/a	+	+
*Ongoing support of local program champions*	The program has been supported by a credible individual from within or external to the organization who is actively involved in facilitating program implementation and sustainability. They champion the need and value of the program in the community, and assist with training, program delivery, creating partnerships, problem solving and monitoring of program activities.	+	+	+	+	n/a	–^b^	n/a	–
*“Exercise post-stroke. Exercise in the community is a priority on our regional work plan and it has been for a number of years. By strategically placing on our work plan and identifying annual activities related to that, that's how I’ve been able to continue with providing this degree of support and involvement in the TIME™ program. As well I have a director who is very understanding and also is a champion of TIME™, sees the value of TIME™ and the benefit of it. I’ve been given that leeway I’ll say.” Regional Coordinator, Site A*									
*“They have a champion within the upper levels of the municipality who believe in these kinds of programs and see the value of these kinds of programs and are willing to explain to the management that you’re not necessarily going to get a lot of numbers and understand that if you’re just kind of breaking even, it's okay. These are not meant to be money makers. But instead they’re providing a service to the community, fulfilling their mandate in terms of being accessible and open to all members of the community.” Regional Coordinator, Site A*									
7. **Ongoing involvement of motivated and experienced staff**	Program staff have previous experience working with individuals with mobility challenges and/or are motivated to work with them. Program staff are encouraged to remain involved by positive changes observed in the participants resulting from program participation and the development of trust and social relationships with these participants over time.	+	+	+	+	n/a	–	–	–
*"I really think the importance of instructors really connecting with our participants is such an important part of it. I think that's true of any group fitness program, specifically with the TIME™ program because it's a little more intimate than a traditional fitness class at any gym. You are trying to rehabilitate people back to a level of more mobility and making them feel good about other aspects of their lives.” Fitness Instructor, Site A*									
*“I think the experience and the skills our staff have. It's just the training that our staff have. It's intense. Well not intense but we ensure that the instructors that run the program are well trained.” Fitness Instructor, Site B*									
*“I think maybe it wasn't the area that some people wanted to work in. So yes, I think the demographics, the type of program, I think some of the team players here and it's not a criticism at all, it's just something they didn't want to do. I don't think all personal trainers or fitness instructors gravitate towards those medical conditions if you will.” Program Instructor, Site H*									

+: Participants identified condition as facilitating sustainability; −: Participants identified condition as deterring sustainability. n/a indicates the condition was not mentioned at a site.

^a^ Competing programs did not affect sustainability.

^b^ Program champion present on initial implementation; however, relationship discontinued.

Recognizing the conditions that existed, program staff in some of the sites attempted to take actions to bolster the favorable conditions or resolve challenging conditions. We refer to these actions as strategies. With the sustained programs, the strategies employed were perceived to be effective in facilitating program continuity and sustainability. In contrast, in sites where program discontinued, staff efforts to influence sustainability failed to prevent discontinuation of the program. Table 5 describes the strategies with supporting quotes and the extent to which the strategies were employed at each site. Reflecting on their experiences, participants shared some recommendations on how other organizations can plan for sustainability that are summarized in Table 6.

**Table 5:** Strategies employed by sites to overcome challenges or optimize program sustainability.

Strategy *Associated condition	Description	Use of Strategy across Sites							
		A	B	C	D	E	F	G	H
1. Organization assigns dedicated resources as needed to ensure smooth ongoing program delivery *Adequate organizational resources	The resources required to deliver the program (e.g., room/space, equipment, time and staff) were consistently available and allocated to the program. When required, the organization had prepared for and was able to re-direct additional resources (additional trained instructors, volunteers, additional time for a second program) to meet the demands of the program.	+	+	+	+	–	–	–	–
*“Our centre is very busy. Where we were providing the program was in the gymnasium and the gymnasium had been asked to use for pickle ball, kids summer camp, wheelchair basketball, so there were a number of other programs that were needing the space. So instead of having a large gymnasium where I and the volunteers could keep an eye on everyone in the group, we were put into two separate rooms. So half of the group was in one room doing one thing, half of the group was in another room and then we would switch. That was not ideal.” Fitness Instructor, Site G*									
2. **Organization assigns a dedicated individual to creating program awareness in the community through marketing, outreach, relationship building, to enhance participant enrollment**	Organization identified and assigned an individual responsible for developing partnerships with healthcare offices and hospitals and creating pathways of referrals. This individual is also tasked with increasing awareness of program in the community through media (radio, television), events, or established networks and partnerships.	+	+	+	n/a	–	–	–	+/-^a^
*Adequate organizational resources									
*Initial and ongoing presence of supportive partnerships									
*“Well it's a lot to do with getting the word out and [coordinator] goes to our hospital and recruits participants or informs them what our services offer and the different programs that we offer to social workers at the hospital. So we get a lot of participants that way.” Fitness Instructor, Site B*									
*“Because my role is such a busy role with programming for so many other things here and also supervising staff in all the human resources functions and such, the TIME™ program wasn't something we were properly able to market and promote. If we would’ve had somebody that was specifically dedicated to administering the program, it would’ve been easier. It was just more so not having the proper amount of time to promote it and really get the connections going.” Program Coordinator, Site F*									
3. **Organization employs strategies to facilitate and sustain enrollment** *Economic viability of the program	Site employed effective recruitment strategies which included a) promoting referrals through the established partnerships and networks with local hospitals and healthcare offices, b) active recruitment of eligible clients in hospitals by program staff, or c) self-referrals from community residents who are aware of the program.	+	+	+	+	+	+	+	+
*Initial and ongoing need for the program in the community	Some programs offered subsidies to encourage continued enrollment while in other programs staff applied strategies such as potlucks to make the class fun and supportive which encouraged participants to return for subsequent sessions.								
*“The last class is like a potluck. People can bring in things. We’ll all chip in five bucks and we’ll order some pizzas. Having them all sitting down at tables and chatting and all that is a nice experience. The social aspect I think too definitely for some of them. It's just seeing those familiar faces that brings them back.” Program Coordinator, Site C*									
*“I think the numbers: being able to build those relationships with your healthcare organizations or with your referring agencies so that you get your numbers but also being in a location in which there is a critical mass of people [is critical] to be able to continue the program.” Program Coordinator, Site B*									
*“In terms of funding we do utilize [name of a fund] which has recently come into play. we do have some participants who are in need of financial assistance so it kind of supports them. We kind of have a pot of money to support that. Other than that, we’re a charity so our money comes from donations and our memberships.” Regional Coordinator, Site A*									
*“I would promote the TIME™ program to the physiotherapists working in the day rehab programs or on the integrated units. Hopefully they can refer. Primary care is a tough nut to crack that way. The advanced practice nurses that work with our program probably have stronger connections to primary care so whenever they get an opportunity, would promote the TIME™ program.” Regional Coordinator/Healthcare Partner, Site A*									
4. **Management encourages program growth and continuity and is supportive of program staff**	Organizational management communicates a commitment to continue the program even in times of low enrollment.	+	+	+	+	–	+	–	+
*Alignment of organizational objectives									
*Ongoing involvement of motivated and experienced staff									
*“With other programs that are more common like intermediate, if we have a low registration, we may look into discontinuing that program. But for something like TIME™ even with a lower registration, we continue to run it regardless because it is a very niche program. And for those four or five people that are coming, that's really important for them to get out of the house and to get moving. It's a bigger thing than just looking at how many people are attending in terms of income and all that kind of stuff.” Program Coordinator, Site D*									
5. Program partners engage to improve enrollment, program delivery and networks *Initial and ongoing presence of supportive partnerships	Healthcare partners and program champions capitalize on pre-existing relationships to generate referrals to the program and establish connections to other complementary programs in the community. In one site, the healthcare partner trained additional instructors to accommodate instructor absence and/or turnover. Importantly their role was funded by the regional rehabilitation program which reduced the financial strain on the program.	*+*	*+*	*+*	n/a	n/a	n/a	*–*	n/a
*“"Well I guess I have the luxury of a role that's kind of nimble and flexible on our annual work plan and the priorities of our region. Exercise is a priority. Exercise post-stroke. Exercise in the community is a priority on our regional work plan and it has been for a number of years. By strategically placing on our work plan and identifying annual activities related to that, that's how I’ve been able to continue with providing this degree of support and involvement in the TIME™ program. As well I have a director who is very understanding and also is a champion of time, sees the value of time and the benefit of it. I’ve been given that leeway I’ll say.” Regional Stroke Coordinator, Site A*									
6. Program staff and management actively fundraise to support program activities in the long term *Economic viability of the program	The organizations undertook certain actions to gather funds to support the program financially. In one case, the organization engaged in fundraising, grant writing, and donation drives to be able to offer program at a subsidized rate to those in need. In a second site, the program manager conducted regular and thorough program evaluations to demonstrate effectiveness and impact to ensure continued funding and demand additional financial support from the local health agency. This allowed the organization to provide two rounds of programming at no cost to the participants.	*+*	*+*	n/a	n/a	n/a	n/a	n/a	n/a
*“Our funding is permanent. We have been lobbying for additional funding. We do have waitlists in our programs and we do want to offer it to more people, so that part we would want to lobby for more funding.” Program Manager, Site B*									
7. **Organization uses membership and/or program registration fees to help fund program activities** *Economic viability	In the absence of dedicated funding, or to supplement available funds, organizations charged membership and/or registration fees to help cover the cost of equipment, space, and instructor time. Some organizations offered subsidies to reduce the cost to participants in need.	*+*	n/a	*+*	*+*	*+*	*+*	*+* ^b^	*+*
*“Well, being with the City I think we basically want to at least break even on most of our programs. That's how it works. We have our targets. If we’re creating a new program idea then we have to get approval and stuff like that but there are budget restraints. Even getting new equipment and such for certain programs and TIME™ does have different equipment than our other programs.” Fitness Instructor, Site C*									
8. Program managers/coordinators reduce the number of instructors and/or rely on volunteers per class to reduce program related costs *Economic viability *Availability of resources	In an attempt to compensate for poor cost recovery due to low enrollment, some programs reduced the number of instructors teaching each class to reduce the ongoing cost associated with program delivery. In other programs, the lead instructor was supported by volunteers rather than trained and experienced instructors to reduce cost without affecting the recommended participant instructor ratio.	n/a	n/a	n/a	n/a	n/a	*-*	*-*	n/a
*“I think it was just getting to the point where it was becoming almost like a personal training session and it wasn't useful time for myself or my staff. What would happen was I would alternate with one of my full-time staff in teaching the program. And so she would teach a day and then I would teach a day and then it would just go like that because we never had enough people to warrant having both of us there.” Program Instructor, Site F*									

+: Indicates that the strategy was employed and led to positive results for the site; –: Indicates that the strategy was employed and did not result in a positive result. n/a: Indicates that the site did not employ this strategy.

^a^ The program manager networked with local healthcare professionals to set up referral pathways, but this was not continued due to competing responsibilities.

^b^ The program was initially offered at no cost to members of the recreation organization, as well as non-members. However, in an attempt to recover program costs, subsequently participants were required to purchase a membership to enroll in the program.

**Table 6:** Recommendations to promote program sustainability from participating sites.

Domain	Recommendation	Source
**Fitness instructor staffing**	1. **Ensure adequate and accessible instructor training:** Instructor training needs to emphasize what to expect when working with people that typically present to TIME™ programs (managing abilities and limitations, how to give individualized attention) to increase their confidence and comfort, as trainers do not receive this knowledge through general fitness training or even training focused on working with older adults. If training sessions are accessible (e.g., offered at the site), more staff and volunteers may be willing to participate in training, providing a better instructor supply to buffer against possible turnover. 2. **Recruit instructors that are motivated to work with people with disabilities:** Programs need to ensure that instructors are committed to being involved with TIME™ long-term and are interested in working with the TIME™ target population. Instructor motivation and commitment to the program help maintain consistency in instruction for the program participants, which allows to build trust and learn how to support them best. This, in turn, may increase class cohesiveness and keep the participants coming back.	Sustained sites: A Discontinued sites: E, F, H
**Participant enrollment**	1. **Invest time and resources towards community outreach and marketing:** Programs need to devote time and financial resources to market the program in the community. Marketing through flyers and recreation guides alone may be insufficient. Using local mass media (e.g., newspaper or radio ads) and sharing anonymized participant stories may generate interest among other people with similar lived experiences. Programs also need to understand that enrollment may happen in waves and low enrollment numbers should not deter continued program promotion. 2. **Develop a network of local healthcare partners:** General marketing to local healthcare settings (e.g., doctor offices and hospitals that serve patients with neurological or neuromuscular conditions) may not be sufficient to provide consistent referrals into the TIME™ program. Programs need to establish partnerships with these settings by making a case for how this partnership may be mutually beneficial (e.g., improving patient flow, offloading rehab resources). Partners need to understand how the TIME™ program may benefit their patients to identify suitable participants. These relationships need to be established early, ideally prior to program implementation, and sustained. 3. **Assign a program administrator to perform active outreach:** Active outreach and patient recruitment, information sharing with other programs, and program coordination and administration are time-consuming tasks that require a separate staff role (cannot be an add-on for staff serving in other capacities or for volunteers). 4. **Balance affordability and cost-recovery when setting membership and program fees:** Programs should aim to minimize cost barriers to participants through affordable pricing and subsidies, while balancing the program input and operating costs.	Sustained sites: A, B, D Discontinued sites: E, F, G
**TIME™ community of practice**	1. **Establish a network of TIME™ programs:** New programs need to seek out opportunities to learn best practices from successful and experienced TIME™ programs (e.g., through site visits, program surveys, or regular meetings) about how to work with people with disabilities, how to implement the exercises, how to establish local partnerships, and how to run, price, and market the program, in the context of the size and location of the program.	Sustained sites: A, B Discontinued sites: F

Based on the common conditions influencing program sustainability and the outcomes of the strategies employed by the sites where programs sustained and discontinued, we proposed a series of questions outlined in Table 7 that providers of new or existing programs may ask themselves to foresee challenges and create an environment conducive to program sustainability.

**Table 7:** Questions to consider to understand the potential for program sustainability.

Question	Rationale
Is there an initial and ongoing need for the CBEP-HCP in the community?	Programs were sustained in areas where there was a demonstrated need for the program in the community.
Is there an initial and ongoing alignment between the CBEP-HCP and organizational goals?	Alignment between program and organizational goals was seen for sustained and discontinued sites, and was deemed necessary to continue offering the program.
Is there a CBEP-HCP champion (internal or external) who can support program implementation and ongoing delivery?	Presence of a program champion was considered a positive feature by sustained programs and as a lacking feature by discontinued sites.
Are program resources (equipment, staff, volunteers) available and will they continue to remain available?	Discontinued programs experienced uncertainty regarding program resources or unavailability of resources required to deliver the program.
Do program instructors have or intend to obtain experience working with individuals with disability?	Sites with inexperienced instructors reported challenges and low motivation to continue working with this population.
Are there sources for participant referral from the community? Can a referral system be developed?	Absence of a secure and robust referral pathway was a major challenge reported by discontinued programs which were terminated due to low participant numbers.
Is there an individual dedicated to oversee participant recruitment efforts?	Managers at discontinued sites reported a need for a separate individual to manage program recruitment.
Can the program obtain secure and dedicated funding?	The availability of complete or partial funding supported program sustainability at two sites.
In the absence of dedicated program funding, what will cost recovery depend on?	When the cost recovery depended on program fees paid by participants, the program sustainability was impacted by participant enrollment numbers.

## Discussion

To our knowledge, this is the first study of its kind to examine the sustainability of CBEP-HCPs that are implemented due to the interest of and initiatives of local organizational management, without guaranteed funding support from the program developers, researchers, local governments, or the healthcare system. Findings highlight factors that, individually and through complex interplays, contribute to the sustainability or discontinuation of a CBEP-HCP. Results suggest that the sustainability of an CBEP-HCP depends on the initial and continued presence of (1) alignment of program and organizational goals, (2) perceived need for program in the community, (3) presence of supportive partners and partnerships to create a referral pathway and increase awareness of program in the community, (4) presence of an experienced and motivated team of individuals involved in program implementation and delivery, and (5) organizational capacity, resources, and funding to support, promote and sustain the program.

The majority of existing research in the area of health program sustainability surround programs related to mental health, addiction, primary and acute care settings, discusses medical interventions or education programs. These programs are either led by researchers as a part of a research study or by government/local authorities as a part of health promotion programs and are supported by ear-marked funding (45). While the findings of this study align with previous research around sustainability of health programs in the community (45–48), the complex interplays we observed during case and cross-case analysis have not been discussed before for programs outside of the healthcare setting.

### Factors influencing sustainability are interrelated and their influence can be modified by purposive action

Sustainability literature divides the factors influencing sustainability into certain sub-groups. Shediac–Rizkallah and Bone (23) model divided the factors broadly into elements within inner settings, outer settings, and program design factors; while more recent reviews such as by Lennox et al. (47) divided them into more distinctive categories such as initiative design and delivery, negotiating initiative process, people involved, resources, organizational setting, and the external environment. However, our study reveals that these factors are in fact not siloed. Rather, the sustainability of a program can be better explained by understanding the conditions created by interaction between these elements, as well as the dynamic influences of the actions taken by the program staff to boost positive elements and mitigate challenge.

Together the characteristics of the inner setting, outer setting and program design interact and influence each other to create an environment where a program thrives and grows or fails to achieve expectations and naturally discontinues or is terminated. For example, the environment for the sustained program can be described as one where the program staff support and encourage the allocation of resources towards a program for which there is an ongoing need in the community. The staff are in turn supported by partnerships and networks that provide ongoing assistance to program delivery. They employed strategies such as fundraising to financially support the program, building networks with a source of participants to promote referral, and providing subsidies to encourage participation.

With discontinued programs, challenging conditions were aggravated by unsuccessful strategies. For example, poor program enrollment due to the presence of competing programs led to reduced motivation among program staff and poor cost recovery. Strategies such as program promotion or reducing number of staff to minimize costs were not sufficient to revive the program and led to program termination. Authors (49, 50) have previously discussed the interaction of these factors but this is the first study to demonstrate the nature of the interaction and the resulting impact of program sustainability in real-world cases.

Our study also expanded on the descriptions (45) of some of the factors thought to influence sustainability from a community-based exercise program perspective. In the case of CBEP-HCPs like the TIME™ program, which are designed for a specific sub-group of individuals, program need is not only reflected in the enrollment of new participants but also the reenrollment of existing participants. In this case, the ability to remain involved for a prolonged period is important to the participants who often require long-term participation to make and maintain health gains and prevent deconditioning. From an organizational standpoint, need for the program in the community also includes the absence of competing programs and the ability of the program to add value (51, 52) – either through alignment with the organizational vision or by creating opportunities to expand their client base.

The need for the program must be complemented by a pathway that connects those who need the program to the program. Both sustained and discontinued programs underscored the need for secure and consistent sources that can refer participants to the program, and for an individual who is responsible for developing networks with these sources of potential participants. For this role, three of the four sustained programs relied on a program staff (healthcare partner, or program coordinator) who had connections and experience in both healthcare and community setting and were able to bridge the gap between the two. Referred to as a boundary spanner (53), these individuals use their connections and presence within both settings to increase awareness of program presence among healthcare professionals and potential participants. When facilitated by an individual in a boundary spanning role, there is an increase in the referrals from important, long-standing sources of clients such as hospitals, community healthcare centres (54, 55) compared to advertisements or marketing. When present, program champions - individuals who believe in and support the program and its potential for impact-helped to increase organizational, and community buy-in for the program, support training, aid in resolving challenges and maintain motivation (56, 57).

Results showed that for a program that involves long-term clients, having experienced and motivated instructors facilitates participant retention. Participants, especially individuals with disabilities, rely on developing long-standing associations with instructors who become aware of the participants’ needs and abilities, and can develop a sense of trust over time (58). Experienced and motivated instructors are also important from a program delivery standpoint as not many instructors have experience and willingness to work with clients who are at a risk for falls or have disabilities (22, 58, 59). As seen in the sustained programs, their commitment to affect positive change motivates them to remain involved in the program thereby reduces staff turnover and costs associated with training and employing new instructors. Similarly, as was reflective in the sustained and discontinued programs, the capacity to allocate supportive resources (volunteers, appropriate space, and equipment) is important to keep the instructors engaged and motivated.

Resource availability is inextricably connected to the availability of funds to support program implementation and delivery. Funding is a complex factor as it depends on the type of organization (private vs. municipal vs. charity), their business model (for-profit vs. not-for-profit), their source of funds (fee-for-service, donations, federal grants etc.), and the assurance of ongoing availability funds for the program. As seen in this study, the availability of ear-marked funding for the program provided a secure foundation for two sustained programs allowing them to engage more staff, offer incentives for participation, and dedicate resources towards the program. The importance of financial support for sustainability while not novel, assumes greater significance when program implementation is initiated at grassroot levels rather than those driven by academic research or government initiatives. Organizations can benefit from resources that indicate the costs involved in program implementation to help with planning for program implementation and continued delivery (60).

### The need to plan for sustainability

The ultimate aim of program sustainability is to ensure that the health benefits to the target population are maintained and that the program is continually accessible to existing and new participants. In the absence of other alternatives, discontinuation of effective programs may result in participants discontinuing engaging in exercises due to a fear or adverse effects, or lack of knowledge of how to exercise safely when experiencing balance and mobility limitations (59, 61). The resulting sedentary lifestyle may cause loss of improvements, deterioration in health, and other secondary complications (62–64).

This study demonstrates that program sustainability cannot be guaranteed by the mere presence of positive factors. It depends on the conditions surrounding the program at the time as well as the strategies employed by the program staff to promote positive influences and overcome challenges. These conditions may change over time. Some authors (46, 65) argue that innovations often go through phases of relative stability interspersed with periods of adaptation. For example, program need may be affected by the emergence of a new competing program, or changes in the demographic composition of the community or priorities of the organization/local community.

It is important to note that the discontinued programs did not anticipate challenges at the time of implementation. Regular monitoring of the conditions surrounding the program could allow program staff to prospectively engage corrective strategies when required. Bodkin and Hakami (45) suggest that challenges and opportunities can be identified through SWOT (strength, weakness, opportunities and threats) analyses. An assessment of the environment and the local context surrounding the implementation will reflect what the needs are, what challenges are specific to the local context, and if the needs are already met. If program delivery can be adapted to the needs, then the organization can avoid program discontinuation.

Studies on program sustainability recommended that planning for sustainability should begin early in a program's life cycle (66, 67). Asking questions such as the ones listed in Table 7 will help the planning team foresee and prepare for challenges or barriers. Understanding the experiencing of different organizations may serve as valuable lessons or examples for other teams considering implementing or re-designing a CBEP-HCP. Similarly, existing and future programs could benefit from the development of a community of practice of CBEP-HCPs where program staff could share experiences, resolve challenges and provide support to each other to promote program sustainability, growth and expansion. Future research should focus on identifying potential solutions to the key challenges of recruitment, program funding, and creating supporting partnerships.

### Limitations and considerations

The sites that participated in this study reflect the heterogeneity of the recreation centres in the community including sites run by the local government/municipality, as well as private, for-profit and not-for-profit organizations. All the programs in this study, however, were located in urban or sub-urban centres; the experience of programs in rural centres is missing. This is important as the priorities and needs of rural centres and the resources available may be unique. The participating sites also belonged to two provinces in Canada: Ontario and British Columbia. The outcomes of programs implemented in other provinces may differ based on their priorities and the resources available to them. These factors may impact the transferability of the findings to other regions.

## Conclusion

Sustainability of CBEP-HCPs for people with balance and mobility limitations is influenced by a complex interaction of conditions surrounding the program and the actions taken by the individuals involved in program implementation and delivery. Understanding what the conditions are and how they interact before implementing the program can stimulate corrective actions, where required, to prevent discontinuation of effective CBEP-HCPs for people with balance and mobility limitations.

## Data Availability

Data are not available as participants did not consent to data sharing. Enquiries should be directed to the corresponding author.
